# Early initiation of combined therapy in severely immunocompromised patients with COVID-19: a retrospective cohort study

**DOI:** 10.1186/s12879-024-09466-y

**Published:** 2024-06-06

**Authors:** Salvatore Rotundo, Lavinia Berardelli, Sara Gullì, Valentina La Gamba, Rosaria Lionello, Alessandro Russo, Enrico Maria Trecarichi, Carlo Torti

**Affiliations:** 1 Dipartimento di Scienze Mediche e Chirurgiche, Università “Magna Graecia”, Catanzaro, Italy; 2 Unità Operativa Complessa di Malattie Infettive e Tropicali, Azienda Ospedaliero-Universitaria “R. Dulbecco”, Catanzaro, Italy; 3 Dipartimento di Scienze di Laboratorio e Infettivologiche, Fondazione Policlinico Universitario Agostino Gemelli IRCCS, Rome, Italy; 4 Dipartimento di Sicurezza e Bioetica, Università Cattolica del Sacro Cuore, Rome, Italy

**Keywords:** SARS-CoV-2, COVID-19, Immunocompromised, Combined therapy, Antivirals, Monoclonal antibodies

## Abstract

This single-centre retrospective cohort study reports on the results of a descriptive (non-comparative) retrospective cohort study of early initiation of antivirals and combined monoclonal antibody therapy (mAbs) in 48 severely immunocompromised patients with COVID-19. The study assessed the outcomes and the duration of viral shedding. The patients started early combined therapy (ECT) a median of 2 days (interquartile range [IQR]: 1–3 days) after the diagnosis of SARS-CoV-2 infection. Except for 1 patient who died due COVID-19-related respiratory failure, patients had their first negative nasopharyngeal swab result after a median of 11 days (IQR: 6–17 days) after starting combined therapy. There were no reports of severe side effects. During a follow-up period of 512 days (interquartile range [IQR]: 413–575 days), 6 patients (12.5%) died and 16 (33.3%) were admitted to hospital. Moreover, 12 patients (25%) were diagnosed with SARS-CoV-2 reinfection a median of 245 days (IQR: 138–401 days) after starting combined treatment. No relapses were reported. Although there was no comparison group, these results compare favourably with the outcomes of severely immunocompromised patients with COVID-19 reported in the literature.

## Background

In severely immunocompromised patients, untreated COVID-19 is characterized by high rates of negative outcomes such as death and relapse [1]. Although early monotherapy with neutralizing monoclonal antibodies (mAbs) or antiviral agents against SARS-CoV-2 is recommended to prevent unfavourable outcomes in immunocompromised patients, the available studies suggest that complication rates remain high [2–6].

Therefore, COVID-19 remains a “sword of Damocles” for severely immunocompromised patients, and recent guidelines recommend longer treatment or additional courses of antivirals in patients with prolonged, symptomatic COVID-19 [7]. Several authors have reported the use of combined therapy in immunocompromised patients, but the incidence of unfavourable outcomes such as clinical worsening [4], relapse [6], and death [3, 4] remains high. Moreover, longer courses of treatment and combined therapy with 2 antivirals and mAbs could be unsafe. For example, both Gentile et al. [4] and Mikulska et al. [3] reported severe side effects in 9.1% of patients.

We previously proposed an algorithm for the treatment of outpatients with COVID-19 who were referred to a treatment centre for frail patients with COVID-19 in the Calabria region of Italy considering their comorbidities and vaccinal status [2].

In this study, we focused on severely immunocompromised COVID-19 patients over an extended follow-up period, aiming to explore the safety and efficacy of early combined therapy (ECT) involving both antivirals and monoclonal antibody therapy. Herein, we reported both clinical (i.e., death, hospital admission and relapse) and virological outcomes.

## Methods

**Fig. 1:**
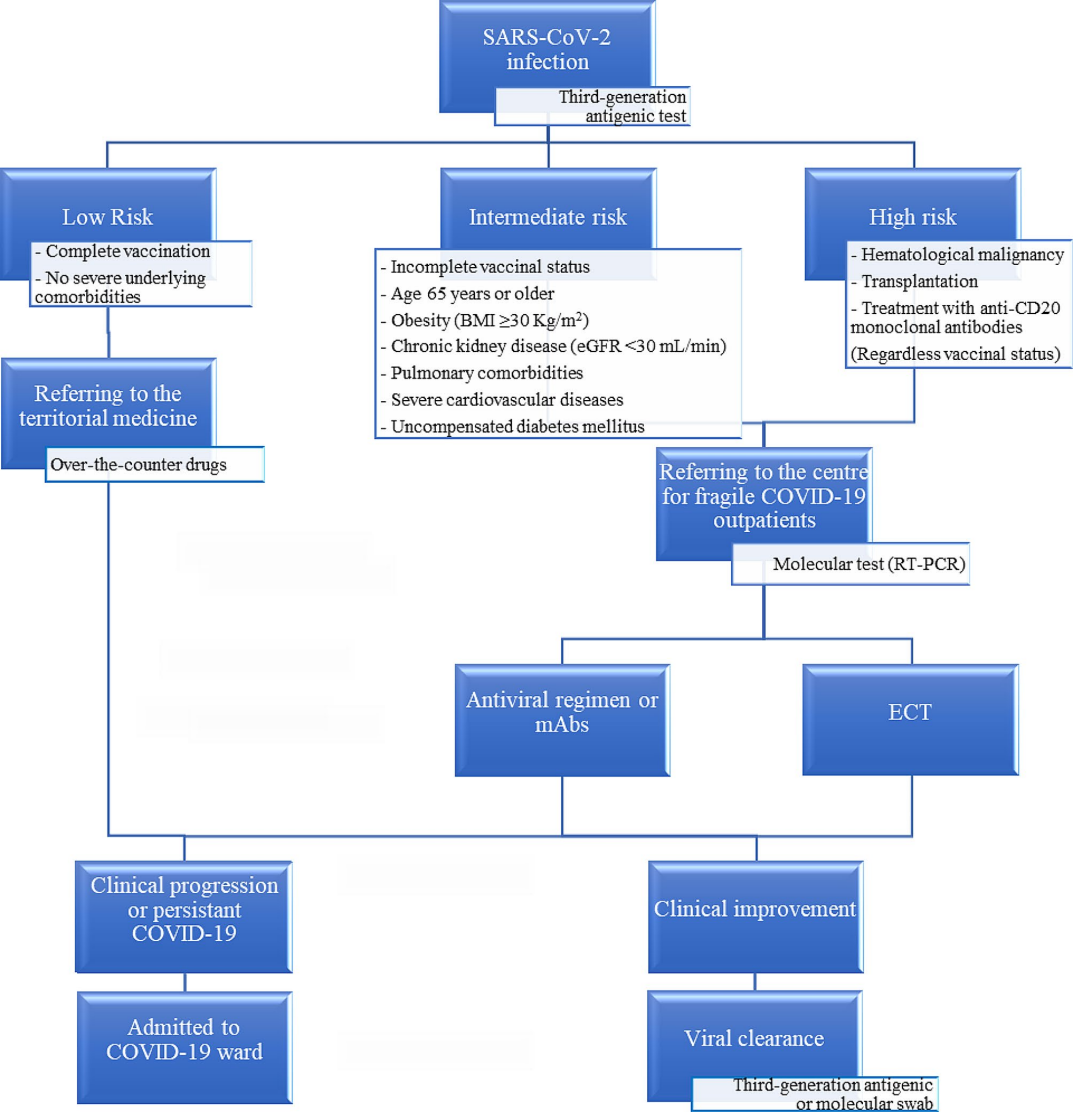
Flowchart illustrating the risk assessment for severe COVID-19 conducted by our facility for COVID-19 outpatients. Only patients who received early combined therapy (ECT) were included in this study. BMI: body mass index; eGFR: estimated glomerular filtration rate. RT-PCR: real-time polymerase chain reaction; mAbs: neutralizing monoclonal antibodies against SARS-CoV-2

We conducted a single-centre, retrospective study including consecutive adult outpatients with major immunocompromising conditions (i.e., hematological malignancy, transplantation or treatment with anti-CD20 monoclonal antibodies and COVID-19) from January 1, 2022, to January 31, 2023. In accordance with the regional health system, these patients underwent third-generation antigenic swab testing for SARS-CoV-2 detection through local medical territorial services, including COVID-19-dedicated territorial physicians or patients’ primary care physicians (Fig. 1).

After initial testing, patients deemed to be at low risk of severe COVID-19 were provided with over-the-counter medications (e.g., nonsteroidal anti-inflammatory drugs, inhaled corticosteroids) and were overseen by local medical territorial services, while those classified as having intermediate to high risk were referred to our specialized centre. Our facility focuses on providing tailored care for fragile COVID-19 patients [2]. SARS-CoV-2 infection diagnosis was confirmed using real-time polymerase chain reaction (RT-PCR, GeneFinder COVID-19 Plus RealAmp Kit, Elitech Group) targeting the RNA-dependent RNA polymerase (RdRP) gene, the envelope (E) gene, and the nucleocapsid (N) gene of SARS-CoV-2. According to literature, a cycle threshold (Ct) value lower than 35 indicated a confirmed positive test result [8]. For confirmed cases, a clinical assessment of the patient’s risk for progression to severe COVID-19 was conducted.

Patients assessed to be at intermediate risk of developing severe COVID-19 were provided with a single-agent COVID-19 early therapy consisting of either antiviral regimen or mAbs. Only patients deemed to be at high risk of severe disease received ECT which included both mAbs and an antiviral regimen. This therapy was initiated simultaneously on the day of SARS-CoV-2 infection confirmation via molecular test. Only these patients were eligible for inclusion in this study.

According to the protocol [2], each patient received ECT against SARS-CoV-2 that included both mAbs (sotrovimab, casirivimab/imdevimab or tixagevimab/cilgavimab), selected for their likely effectiveness against the spread of local SARS-CoV-2 variants, and an antiviral agent (intravenous remdesivir for 3 days or 5 days of oral nirmatrelvir/ritonavir or molnupiravir) selected in accordance with their availability, feasibility of administering parenteral treatment, and potential for drug-drug interactions, as indicated by international guidelines [7]. Patients were actively monitored until the time of the first nasopharyngeal swab with a negative SARS-CoV-2 result. Outcomes occurring after viral clearance were recorded by performing follow-up phone calls between October 21, 2023, and November 3, 2023, extending the follow-up period to a median of 512 days (interquartile range [IQR]: 413–575 days) from ECT.

## Results

The characteristics of the 48 patients included in the cohort are summarized in Table 1.

**Table 1:** Baseline characteristics of immunocompromised patients who received early combined therapy (ECT) against COVID-19. Data are presented as number (n.) and percentage (%) or median and interquartile range (IQR).

Baseline characteristics	
Patients, n.	48
Age, median (IQR)	58 (45–73)
Male gender, n. (%)	27 (56.2)
BMI, median (IQR)	26.6 (22.3–29.3)
Charlson Comorbidity Index (CCI), median (IQR)	5 (3–8)
Underlying comorbidities	
Hematological malignancy, n. (%)	31 (64.6)
Non-Hodgkin lymphoma, n. (%)	24 (50)
Receiving anti-CD20 monoclonal antibodies, n. (%)	18 (37.5)
Chronic lymphocytic leukemia, n. (%)	7 (14.6)
Hematopoietic stem cell transplant, n. (%)	3 (6.2)
Solid transplant, n. (%)	8 (16.7)
Kidney, n. (%)	7 (14.6)
Liver, n. (%)	1 (2.1)
Autoimmune disorders, n. (%)	7 (14.6)
Requiring anti-CD20 monoclonal antibodies, n. (%)	6 (12.5)
Hypertension, n. (%)	21 (43.7)
Obesity, n. (%)	8 (16.7)
Chronic obstructive pulmonary disease, n. (%)	2 (4.2)
Cirrhosis, n. (%)	1 (2.1)
Diabetes, n. (%)	6 (12.5)
History of stroke or myocardial infarction, n. (%)	7 (14.6)
Chronic kidney disease, n. (%)	9 (18.7)
Solid cancer, n. (%)	5 (10.4)
COVID-19 severity according to National Institute of Health (NIH)	
Asymptomatic, n. (%)	4 (8.3)
Mild, n. (%)	44 (91.7)
Vaccinal status	
Non vaccinated, n. (%)	10 (20.8)
Two doses or less, n. (%)	15 (31.2)
Three doses or more, n. (%)	24 (50)
Cycle threshold (Ct) values	
RNA-dependent RNA polymerase (RdRP), median (IQR)	22 (20–25)
Envelope (E) gene, median (IQR)	20 (16–24)
Nucleocapsid (N) gene, median (IQR)	20 (17–23)
Laboratory findings on day of ECT	
Positive SARS-CoV-2 anti-spike serology, n. (%)	16 (33.3)
White blood cells count, 10^9^ cells/L, median (IQR)	4.42 (3.43–5.55)
Lymphocytes count, 10^9^ cells/L, median (IQR)	0.84 (0.67–1.17)
Interleukin-6, pg/mL, median (IQR)	7 (5–15)
LDH, UI/L, median (IQR)	225 (204–268)
PCR, mg/L, median (IQR)	9 (6–17)
Days from positive nasopharyngeal swab to ECT, median (IQR)	2 (1–3)
Days from onset of symptoms to ECT, median (IQR)	3 (2–5)

**Fig. 2:**
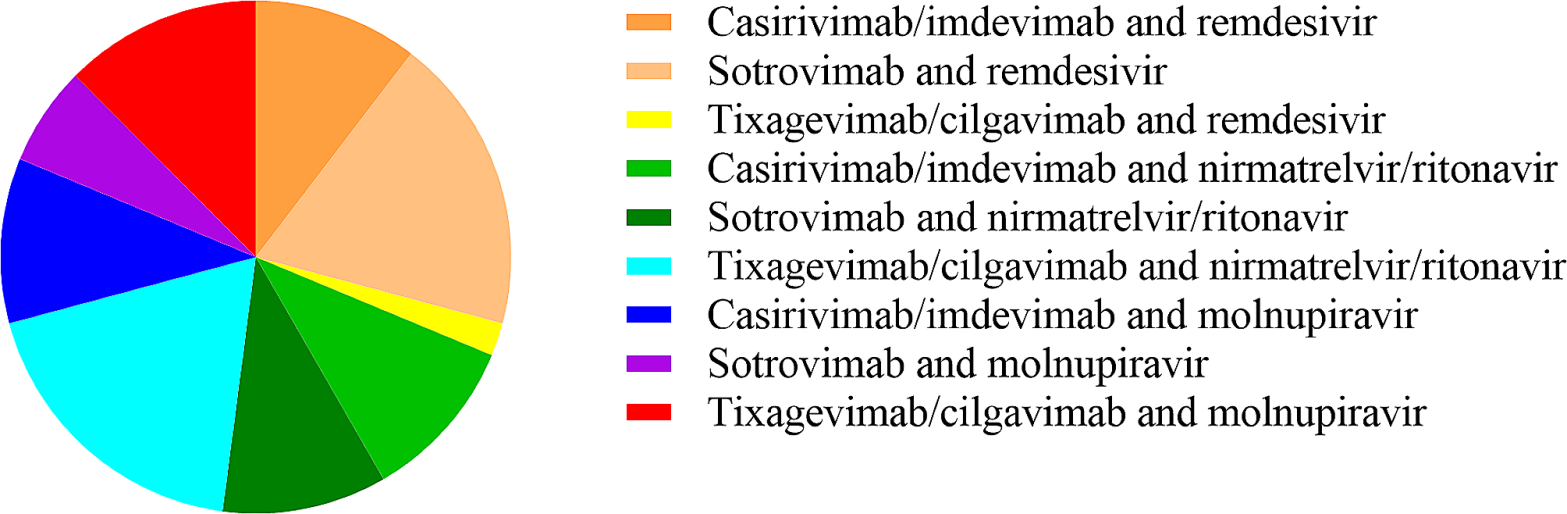
The early combined therapy (ECT) regimens administered to the outpatients included in this study

The majority of patients (44/48, 91.7%) were classified as having mild disease according to the severity scale provided by the National Institutes of Health (NIH) [7]. The remaining patients (4/48, 8.3%) were asymptomatic and tested positive through screening by territorial medical services due to their contact with a COVID-19 case. Therefore, none of these patients were provided with other medications approved for moderate to severe COVID-19 (e.g., systemic corticosteroids, tocilizumab) according to NIH guidelines [7]. The patients started ECT a median of 2 days (IQR: 1–3 days) after SARS-CoV-2 infection was diagnosed. Following the onset of symptoms, patients were referred to the centre for COVID-19 outpatients after a delay of 3 days (IQR: 2–5 days). At this time, confirmatory molecular testing was provided, and ECT was initiated (Fig. 2).

The median Ct values at confirmation of SARS-CoV-2 infection were 22 (IQR: 20–25), 20 (IQR: 16–24), and 20 (IQR: 17–23) for the genes RdRP, E, and N, respectively.

**Fig. 3:**
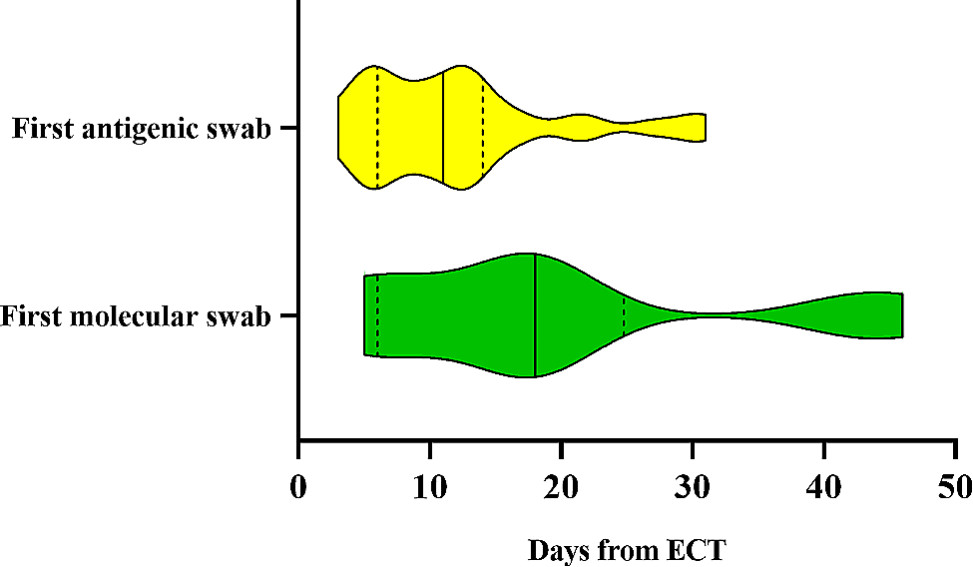
Violin plot representing days from early combined therapy (ECT) to the first negative test. Data are presented as median (solid lines) and interquartile range (dashes lines).

Only 2/48 (4.2%) patients were admitted to the hospital after receiving ECT due to COVID-19 before achieving viral clearance. Both patients had hematological malignancies and were admitted after 3 and 23 days form ECT initiation, respectively. Except for 1 of these patients who died due COVID-19-related respiratory failure after ECT comprising molnupiravir and tixagevimab/cilgavimab, patients had their first negative nasopharyngeal swab result after a median of 11 days (IQR: 6–17 days) after starting ECT. Among survivors, 13/47 (27.7%) were tested with molecular swab and achieved their first negative test results after a median of 18 days (IQR: 6–25 days) from ECT. Conversely, 34/47 (72.3%) patients underwent testing with a third-generation antigenic swab, with negative results obtained after a median period of 11 days (IQR: 6–14 days) from ECT (Fig. 3). No severe side effects were reported.

During the follow-up period subsequent to viral clearance, e5 patients (10.6%) died and 14 (29.9%) were admitted to hospital. Moreover, 12 patients (25.5%) were diagnosed with SARS-CoV-2 reinfection in a median of 245 days (IQR: 138–401 days) after starting ECT. No relapses were reported.

The 5 patients who died after achieving viral clearance were older (median age: 77 years, IQR: 64–82 years) than those who survived (median age: 55 years, IQR: 44–71 years; *p* = 0.04, Mann–Whitney U test), and death occurred in a median of 276 days (154–397 days) after starting ECT. Among the patients who were hospitalised after achieving viral clearance following ECT (14/47, 29.8%), only 1 was admitted due to SARS-CoV-2 reinfection 236 days after the initial infection and recovered after receiving sotrovimab monotherapy. The other patients were admitted to hospital primarily for reasons related to their underlying diseases after a median of 222 days (IQR: 173–242 days). The prevalence of hematologic malignancies was significantly higher in hospitalised patients (13/14, 92.8%) than in those who did not require hospital admission (16/33, 48.5%; *p* = 0.008, Fisher’s exact test).

## Discussion

We applied an innovative strategy aimed at preventing clinical worsening, including death, hospitalization, and relapse, with earlier initiation of combined therapy in severely immunocompromised outpatients with COVID-19. This approach was deemed necessary because neither vaccination alone [9] nor therapies approved for immunocompetent individuals are adequate to sufficiently shield these individuals from the risk of experiencing more severe outcomes [2–6].

In our cohort, a high incidence of adverse clinical outcomes was expected for several reasons. First, a relatively low percentage of our patients received vaccination, with 20.8% having not received any dose of the vaccine. Consequently, a high rate of severe progression of COVID-19 could have been observed in our population [10]. Second, our cohort demonstrated profound immunocompromise, as indicated by the low rate of response to vaccination, with only a third of patients testing positive for SARS-CoV-2 anti-spike antibodies before receiving ECT. This finding aligns with findings from other studies in the literature conducted on patients with hematological malignancies [11], solid organ transplant recipients [12], and those undergoing immunosuppressive therapy [13] which consistently reported incomplete responses to vaccination in these severely immunocompromised patient groups. Third, low Ct values were observed with RT-PCR on molecular swabs for SARS-CoV-2 RNA detection, with a median Ct value below 25 for all genes (RdRP, E, and N) at confirmation of SARS-CoV-2 infection. Importantly, despite pre-analytical factors such as swabbing technique and input volume for nucleic acid extraction as well as variations in assays and gene targets should be considered, as these factors may impact the reliability of Ct values [14], Ct values have been recognized as a surrogate for viral load [14–16] and it was suggested that Ct values may be associated with an increased risk of mortality [16]. Interestingly, Ct values at diagnosis may correlate with an increased likelihood of mortality in patients with hematological malignancies [17]. Fourth, alongside the immunocompromised status, our patients exhibited a notable burden of comorbidities, as assessed by the Charlson Comorbidity Index (CCI) with a median score of 5 (IQR: 3–8).This index serves as a clinically valuable tool for highlighting significant prognostic differences among patient subgroups sharing similar medical diagnoses [18]. This score has emerged as an effective tool for predicting mortality since the onset of the COVID-19 pandemic. Indeed, a metanalysis conducted by Tuty Kuswardhani et al. [19] found that a Charlson Comorbidity Index (CCI) score of 3 or higher was prognostically associated with mortality and a composite outcome including mortality, the need for critical care, severe disease presentation, and mechanical ventilation. Furthermore, each point increase in the CCI is associated with a 16% higher risk of mortality [19]. However, these estimations were derived from studies involving hospitalized patients with COVID-19, and the utility of CCI in predicting outcomes for COVID-19 immunocompromised outpatients has not been validated yet.

Although it is difficult to draw conclusions based on comparisons of different uncontrolled studies, our patients experienced better outcomes than untreated patients [1] and patients treated with monotherapy regimens [2]. In contrast, Lahouati et al. [20] reported rates of hospitalization (4.5%) and death (1.8%) similar to those reported in our study in severely immunocompromised patients, using early initiation of monotherapy after SARS-CoV-2 diagnosis. However, their study [20] was conducted during in the Omicron period, and our patients had a lower vaccination rate than their patients (20.8% vs. 3% were unvaccinated) and probably had more severe immunosuppression (66.7% vs. 16% tested negative for SARS-CoV-2 anti-spike antibody); therefore, our patients could have been expected to experience a worse outcome.

Consistent with the results of our study, Orth et al. [5] found that combined treatment was both safe and effective in prevent prolonged viral shedding in immunocompromised patients, although their results refer to the Omicron wave. Interestingly, the initiation of combined therapy after 5 days after diagnosis was associated with prolonged viral shedding [5]. Triebelhorn et al. [21] recently reported that patients who did not fully respond to SARS-CoV-2 vaccination were more likely to be hospitalized, even when treated with monoclonal antibodies (mAbs). They observed higher hospitalization rates in patients receiving mAbs monotherapy compared to our cohort undergoing ECT (7.3% vs. 4.2%). Moreover, Gentile et al. [4] compared patients treated with combined treatment earlier and later and found that those treated later experienced worse outcomes. These results are consistent with our results, although the study by Gentile et al. [4] had a limited sample size, and both studies lacked a comparison group of patients treated early with monotherapy. Therefore, it is important to conduct appropriate controlled studies to assess whether ECT provides clinical benefits. Further studies should also investigate whether ECT prevents the emergence and accumulation of viral mutations, which is a major risk in immunocompromised individuals [2, 22] because in patients with prolonged symptomatic disease, the emergence of mutations can impair subsequent response to combined treatment. This question is important because the emergence of resistant viruses can lead to their eventual spread at population level [23].

This study has limitations that should be acknowledged. First, the small sample size may limit the generalizability of the findings and we were unable to identify variables associated with hospitalization or death from COVID-19, primarily due to the low occurrence of these outcomes in our cohort. However, the low rates of observed unfavourable outcomes may be attributed to the effectiveness of ECT. Nonetheless, these results are relevant because severely immunocompromised patients experience prolonged viral shedding, relapse, hospitalization, and death [1, 3, 5] despite receiving mAbs or antiviral therapy for SARS-CoV-2 infection [2–4, 6, 21]. Second, the absence of a control group hinders the ability to compare outcomes and assess the true efficacy of the combined therapy approach. However, the strength of this study lies in its focus on a specific population of severely immunocompromised patients, for whom there is a lack of established treatment protocols, especially in the early stages of COVID-19 [7]. Focusing on this high-risk group and evaluating the outcomes of ECT, we provide valuable insights into a previously understudied area of COVID-19 management. Third, while variations in the length of infection prior to the initiation of ECT among patients may indeed introduce confounding factors, they also reflect the real-world setting where patients medical care seeking is delayed before treatment. Importantly, in our centre, we prioritize minimizing the delay between diagnosis and treatment which was 2 days (IQR: 1–3 days) in this cohort severely immunocompromised outpatients with COVID-19. This proactive approach aims to promptly address the needs of this fragile patient population and optimize treatment outcomes.

## Conclusions

In patients with severe immunosuppression, there is a strong rationale for prescribing combined anti-SARS-CoV-2 therapy as soon as possible after diagnosis to prevent prolonged viral replication and the consequent emergence of escaping mutants. The present results provide evidence that ECT appears to be safe and effective in preventing SARS-CoV-2 clinical complications, prolonged viral shedding, and relapse. However, the generalizability of our results may be limited because this is a retrospective, single-center, uncontrolled study. Therefore, prospective studies and controlled trials are urgently required to validate the “early and hard” approach for treating severely immunocompromised patients to inform guidelines from an evidence-based perspective.

## Data Availability

The data that support the findings of this study are available from the corresponding author upon reasonable request.
